# Supplementary material for: Elevation Influences Belowground Biomass Proportion in Forests by Affecting Climatic Factors, Soil Nutrients and Key Leaf Traits
Source: Plants (Basel). 2024 Feb 28;13(5):674. doi: 10.3390/plants13050674 (PMC10935182; doi:10.3390/plants13050674)
Supplement: Supplementary file 1 [file plants-13-00674-s001.zip › supplementary Figure.pdf]

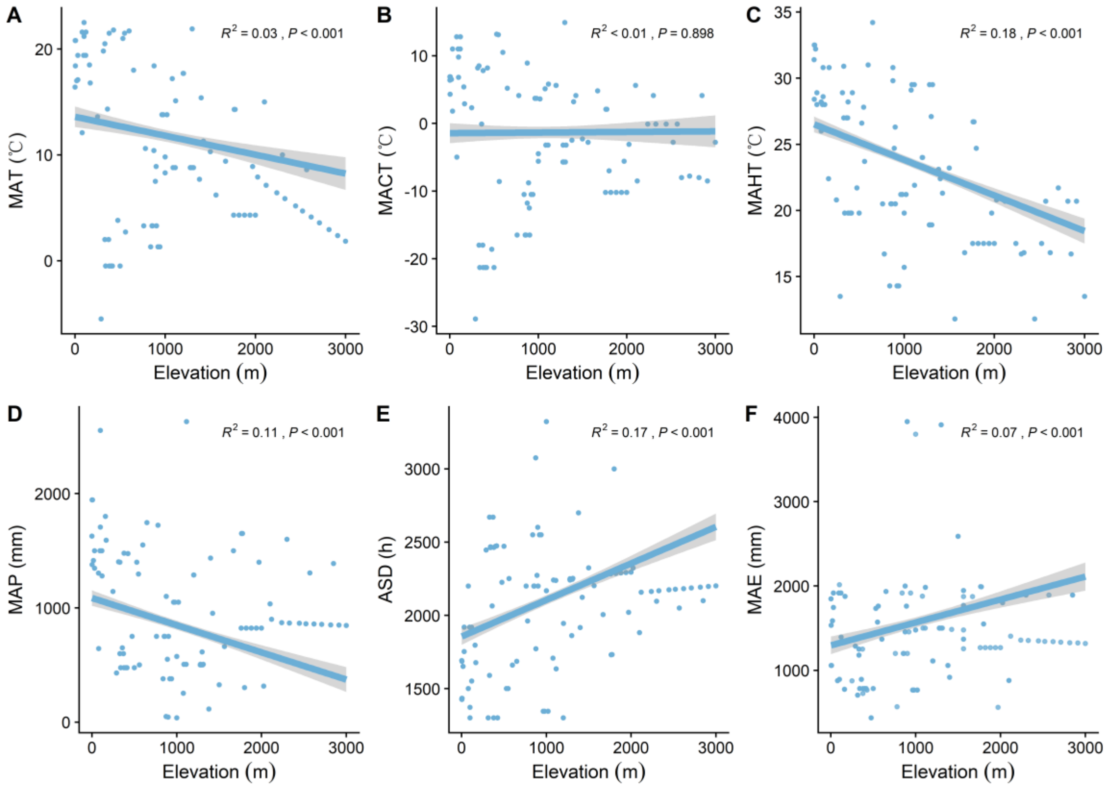

Figure S1 Linear relationship between elevation and climatic factors

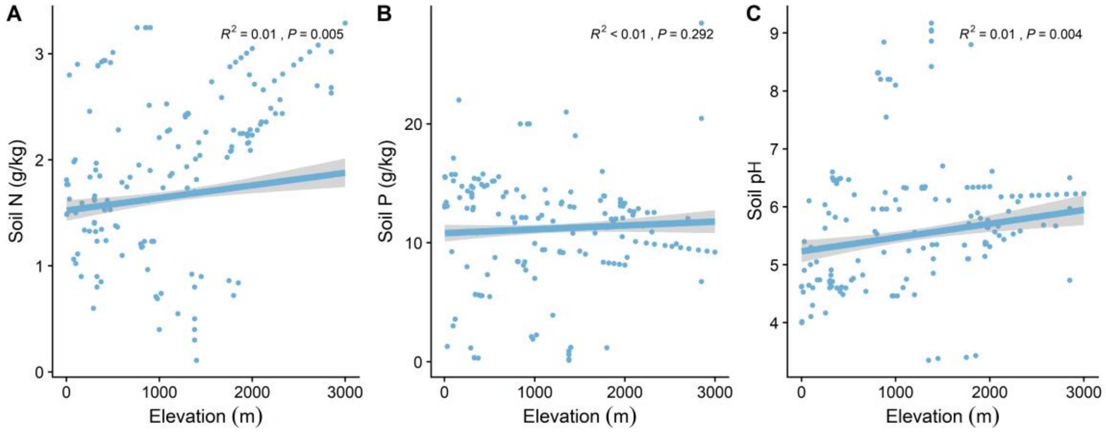

Figure S2 Linear relationship between elevation and soil nutrients

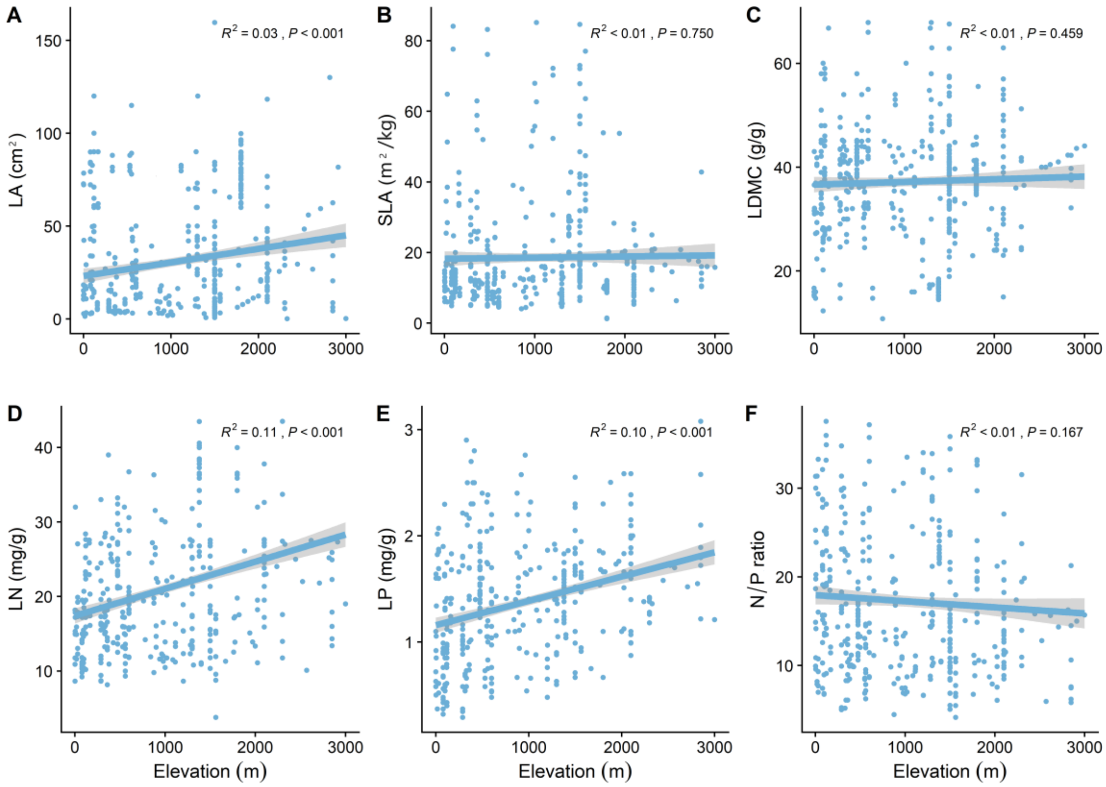

Figure S3 Linear relationship between elevation and leaf functional traits
